# Health Effects of Carotenoids during Pregnancy and Lactation

**DOI:** 10.3390/nu9080838

**Published:** 2017-08-04

**Authors:** Monika A. Zielińska, Aleksandra Wesołowska, Beata Pawlus, Jadwiga Hamułka

**Affiliations:** 1 Department of Human Nutrition, Faculty of Human Nutrition and Consumer Sciences, Warsaw University of Life Science—SGGW, 159c Nowoursynowska St., 02-776 Warsaw, Poland; monika_zielinska@sggw.pl; 2 Laboratory of Human Milk and Lactation Research at Regional Human Milk Bank in Holy Hospital, Department of Neonatology, Medical University of Warsaw, 63A Zwirki i Wigury St., 02-091 Warsaw, Poland; aleksandra.wesolowska@wum.edu.pl; 3 Neonatal Unit, Holy Family Hospital in Warsaw, 25 Madalinskiego St., 02-544 Warsaw, Poland; b.pawlus@szpitalmadalinskiego.pl

**Keywords:** carotenoids, xanthophylls, oxidative stress, pregnancy, lactation, infants

## Abstract

Adequate nutrition is particularly important during pregnancy since it is needed not only for maintaining the health of the mother, but also determines the course of pregnancy and its outcome, fetus development as well as the child’s health after birth and during the later period of life. Data coming from epidemiological and interventions studies support the observation that carotenoids intake provide positive health effects in adults and the elderly population. These health effects are the result of their antioxidant and anti-inflammatory properties. Recent studies have also demonstrated the significant role of carotenoids during pregnancy and infancy. Some studies indicate a correlation between carotenoid status and lower risk of pregnancy pathologies induced by intensified oxidative stress, but results of these investigations are equivocal. Carotenoids have been well studied in relation to their beneficial role in the prevention of preeclampsia. It is currently hypothesized that carotenoids can play an important role in the prevention of preterm birth and intrauterine growth restriction. Carotenoid status in the newborn depends on the nutritional status of the mother, but little is known about the transfer of carotenoids from the mother to the fetus. Carotenoids are among the few nutrients found in breast milk, in which the levels are determined by the mother’s diet. Nutritional status of the newborn directly depends on its diet. Both mix feeding and artificial feeding may cause depletion of carotenoids since infant formulas contain only trace amounts of these compounds. Carotenoids, particularly lutein and zeaxanthin play a significant role in the development of vision and nervous system (among others, they are important for the development of retina as well as energy metabolism and brain electrical activity). Furthermore, more scientific evidence is emerging on the role of carotenoids in the prevention of disorders affecting preterm infants, who are susceptible to oxidative stress, particularly retinopathy of prematurity.

## 1. Introduction

**Figure 1:**
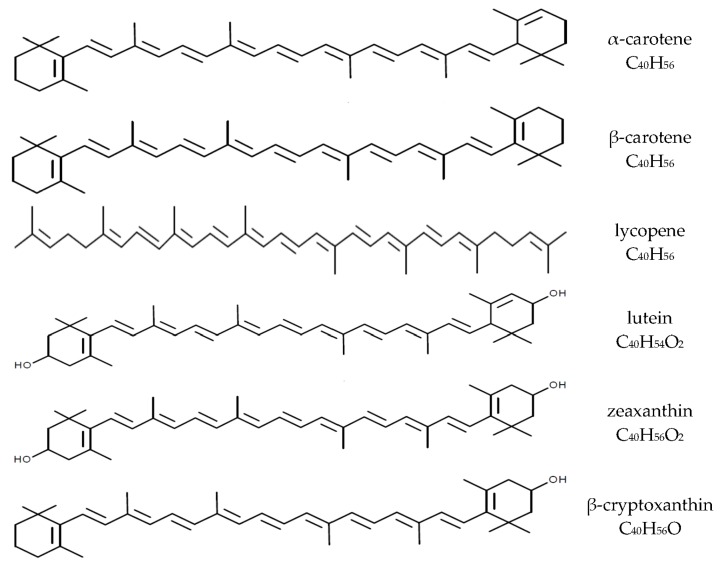
Chemical structures and molecular formulas of selected carotenoids.

Carotenoids are fat-soluble pigments synthesized by plants and some microorganisms. Thus far, more than 700 carotenoids have been identified and belong to groups of carotenes (e.g., β-carotene, α-carotene, and lycopene) as well as their hydroxylated derivatives-xanthophylls (e.g., lutein and zeaxanthin, β-cryptoxanthin, and astaxanthin). About 50 of these carotenoids can be found in the human diet, mainly of plant origin, and some are present in dietary supplements [1,2,3]. Plasma levels of carotenoids are determined by their intakes from the diet, but about 95% of plasma carotenoids are represented by only six compounds: β-carotene, α-carotene, lycopene, and β-cryptoxanthin as well as lutein and zeaxanthin (often analyzed together; Figure 1) [1,3,4]. The nutritional and health effects of carotenoids are due to their multidirectional biological effects in humans, including antioxidant, anti-inflammatory and immunomodulatory properties [1,5,6]. Furthermore, some carotenoids (β-carotene, α-carotene, and β-cryptoxanthin) can be converted to vitamin A in humans, which can contribute to meeting the requirement for this essential vitamin [1,4]. The β-carotene conversion ratio to vitamin A is 12:1 (24:1 for others carotenoids [7]), and is altered by individual’s vitamin A status, food matrices, food preparations and the fat content of a meal [7,8]. The WHO estimates that about 19 million pregnant women in low-income countries are affected by vitamin A deficiency [9]. The importance of β-carotene as a source of vitamin A for pregnant and lactating women has been reviewed by Strobel et al. [10].

Many biological properties of carotenoids help maintain health by decreasing the risk of chronic non-communicable diseases, such as cancer, cardiovascular disease, some eye disorders and age-related decline in cognitive functions, which has been shown in association studies [1,3,4,11,12].

Nutrition during pregnancy is crucial for maternal health, pregnancy outcomes as well as for fetus development and child health both after birth and in later life [13]. Studies have indicated that carotenoids play an important role in pregnancy outcome and in the prevention of many pathologies of pregnancy that are brought about by increase oxidative stress [14,15,16]. In addition, data have shown that carotenoids may help in maintaining optimal health during childhood, including improvement in the development and maintenance processes of vision and cognition [17,18,19,20,21,22,23].

## 2. Pregnancy and Oxidative Stress

Changes in energy metabolism as well as hormonal changes occur during pregnancy. The production of reactive oxygen species (ROS) is increased, which is related to, among others, the functioning of the placenta [24]. During the first trimester, the placenta is not yet connected to maternal circulation and therefore oxygen concentration in the placenta is very low. As a result of this hypoxic state, ROS are generated, which induce the production of factors that regulate proliferation of cells and angiogenesis, including hypoxia-inducible factors (HIF), vascular endothelial growth factor (VEGF) and placental growth factor (PGF) [14,15,24]. Towards the end of the first trimester, maternal circulation within the placenta becomes fully established, which leads to a three-fold increase in oxygen concentration, which in turn raises the level of ROS particularly in the syncytiotrophoblast (fetus part of the placenta that directly participates in metabolic exchange between the maternal blood and that of the fetus). At this time, there is full regulation of the production of HIF-1α as well as the expression of genes encoding antioxidant enzymes, including heme oxygenase 1 and 2 (HO-1 and HO-2), zinc-copper superoxide dismutase (Cu/Zn-SOD), catalase, glutathione peroxidase (GPx) [15,24]. Improper placenta formation, insufficient antioxidant defense as well as increase production of ROS can lead to oxidative stress. Large amounts of ROS cause structural and functional damages to cells and tissues and can act as pro-inflammatory agents, which can lead to many pregnancy complications and abnormalities [15,24]. However, it should be noted that physiological levels of ROS play a crucial regulatory role in female reproduction system, including signaling transduction pathways, e.g., folliculogenesis and embryonic implantation [24]. Carotenoids together with other dietary antioxidants may help in protecting humans against extensive oxidative stress and its debilitating complications [1,3].

## 3. Carotenoids Intake and Their Plasma Levels in Pregnant Women

The intakes of carotenoids by pregnant women have been shown to be variable and depend primarily on the population investigated (Table 1). Results of studies indicate that the consumption of carotenoids is lower among pregnant women who smoke [25,26], younger pregnant women [16] and women who get pregnant in spring and summer (from studies conducted in New Zealand) [27]. The influence of seasonality of the year on the intake of carotenoids may be the result of the differences in the availability of their dietary sources, which are fruits and vegetables, marketing and dietary habits, as well as changes in maternal diet during pregnancy [4,28,29]. In addition, the seasonal variation in carotenoids intake may be explained by differences in its levels in vegetables and fruit between seasons, due to storage or processing changes (both positive and negative) which was summarized by Maiani et al. [4].

The results of the Norwegian Mother and Child Cohort Study (MoBa) confirm that the plasma levels of carotenoids in pregnant women are a strict function of their intakes from fruits and vegetables [30]. The plasma concentrations of carotenoids correlated with the consumption of vegetables (*r* = 0.32; *p* < 0.01), fruits and vegetables together with vegetable juices (*r* = 0.24; *p* < 0.01); the plasma level of α-carotene correlated with the intake of carrots (*r* = 0.50; *p* < 0.01) and cooked vegetables (*r* = 0.39; *p* < 0.01); and lutein with the ingestion of cooked vegetables excluding root and cruciferous vegetables (*r* = 0.30; *p* < 0.01). In the case of β-cryptoxanthin, the highest correlation was found for citrus fruits and their juices (*r* = 0.39; *p* < 0.01) as well as juices in general (*r* = 0.30; *p* < 0.01) [30]. It is worth mentioning that most pregnant women take dietary supplements, which can be a significant source of β-carotene [2,17,25,30]. In the MoBa study, the plasma concentration of carotenoids in women who did not take supplements with carotenoids (*n* = 106) was found to be 1.0 ± 0.50 µmol/L, but was higher in women who used such supplements (*n* = 3) −2.10 ± 0.55 µmol/L [30].

Studies on changes in carotenoid status carried out among pregnant Peruvian women (*n* = 78) [31] as well as pregnant Dutch women (*n* = 140) [32] showed that the plasma concentration of selected carotenoids, particularly lutein, increases by about 40% between the first and third trimester. In both studies, the plasma level of lutein was also found to increase from 0.41 (95% confidence interval (CI) 0.37–0.47) in the first trimester to 0.61 (0.57–0.67) µmol/L in the third trimester (*p* ≤ 0.0001) [31] as well as from 0.48 ± 0.03 to 0.65 ± 0.04 µmol/L (*p* ≤ 0.001) [23]. Simultaneously, the plasma concentration of β-carotene was found to decline by about 20% (*p* ≤ 0.01) in the Dutch study. Unlike tocopherols, the plasma concentration of carotenoids does not proportionally increase with the increase in the level of polyunsaturated fatty acids, which are particularly very prone to oxidative changes [32]. Lower plasma concentration of carotenoids in pregnant women can be caused by smoking [26,33] as well as other non-nutritional factors and lifestyle, such as multiple pregnancies, short interval between pregnancies as well as breastfeeding [34,35]. The intake of carotenoids from dietary sources were analyzed in these studies, therefore it is difficult to state whether the causes of these changes were a result of altered dietary behavior or a function of other physiological determinants.

## 4. Carotenoids and the Prevention of Pathology of Pregnancy

Recent studies have confirmed that oxidative stress can increase the risk of spontaneous abortion and other pathologies of pregnancy, including gestational diabetes mellitus (GDM), preeclampsia, pregnancy-induced hypertension and intrauterine growth restriction (IUGR) as well as preterm birth [15,24]. The role of oxidative stress and protective action of carotenoids against preeclampsia, which affects about 3–5% pregnant women have been well documented [36].

Pregnant women with preeclampsia have been found to have an increase in parameters of oxidative stress (in the placenta, maternal circulation and exhaled air) and inflammation as well as lower antioxidant status and endogenous antioxidants, including carotenoids in the plasma and placenta [37,38,39,40,41,42,43,44,45,46,47]. Low level of endogenous antioxidants can be noticed before the development of preeclampsia. A study conducted by Cohen et al. [48] revealed that the risk of the occurrence of preeclampsia decreases with the increase in plasma concentration of lutein (odds ratio (OR) 0.60, 95% CI 0.46–0.77). However, separate analyses of cases of early preeclampsia (<34 Hbd (weeks of pregnancy)) showed higher protective role of carotenoids with the exception of lycopene and tocopherol in compared with late-onset. Discrepancies in carotenoid’s effect on two other preeclampsia types may be caused by differences in its etiology pathways [48]. Lower plasma concentrations of α- and β-carotene (by 45% and 53%, respectively) have been found in women with type 1 diabetes mellitus and preeclampsia, which developed during the third trimester of pregnancy as compared with women with diabetes but without preeclampsia; no differences were noticed for the plasma levels of lutein, lycopene and α-tocopherol [49]. Studies conducted in Peru (*n* = 304) did not show any risk of the occurrence of preeclampsia in relation to serum level of carotenoids (details about this study and others from these section are presented Table S1 Supplementary Materials) [50]. Lack of statistical significant in this study may be caused by differences between gestational age at blood collection (36.0 ± 0.6 vs. 37.3 ± 0.3, *p* < 0.05), as well failure to adjust for gestational age at blood collection and collecting maternal blood samples after preeclampsia diagnosis [50]. However, studies carried out in Zimbabwe (*n* = 359) demonstrated a decrease risk, even by 50%, for the preeclampsia in women with higher blood levels of β-carotene (OR 0.50, 95% CI 0.25–1.00) [41]. Some researchers have also looked into how dietary habits impact on the risk for the occurrence of preeclampsia. The Norwegian Mother and Child Cohort Study (MoBa), which was conducted in 23,433 women with first pregnancy from which 5.4% developed preeclampsia showed that women who consumed a well-balanced diet rich in plant-based foods (vegetables, vegetable oils, olive oil, fruits, rice) were characterized by lower risk for the development of preeclampsia (OR 0.72, 95% CI 0.62–0.85) [51]. Simultaneously, a systematic literature review and meta-analysis of 10 studies have confirmed the positive role of the consumption of large amounts of fruits and vegetables in protecting against preeclampsia [52]. Usually, confounding factors used for adjusting the results are maternal age, BMI, smoking socioeconomic status, as well total energy intake, however factors such as gestational age, physical activity and hypertensive disorders of pregnancy in earlier pregnancy are seldom [52]. Nonetheless, using these factors are very important for results adjusted, due to the association between healthier diet (e.g., higher vegetables and fruits consumption) and healthier lifestyle (e.g., non-smoking), which may be pro-health factor in itself [53,54]. There are scarce data from observational studies showing the link between the intakes of dietary carotenoids and the development of preeclampsia; however, there are three clinical studies conducted on a small group of women in India. In these studies, investigators analyzed the influence of the intake of lycopene supplements in the dosage of 2 mg/day up to the second trimester of pregnancy. The results of one of the studies showed a very weak decrease risk for the development of preeclampsia (8.6% vs. 17.7% cases, *p* = 0.043) [55], while the two other studies did not confirm such an association [56,57]. Both studies showing no effects of carotenoids had small sample size and were conducted on samples with different health status (*n* = 54, high preeclampsia risk [57] and *n* = 159, low preeclampsia risk [56], and were generally characterized by low quality [16]. Study conducted in low preeclampsia risk group also found adverse effects of lycopene supplementation on incidence of preterm labor (10.4% vs. 1.2%, *p* = 0.02) and low birth weight (22.1% vs. 36.6%, *p* = 0.05) [56]. These effects may be caused by the possibility of lycopene oxidation, which may occur in some condition e.g., exposure on cigarette smoke what was found on β-carotene, however characteristic of study population is limited and there is lack of data about cigarette smoking or environmental exposition on cigarette smoke [56,58]. Meta-analysis conducted by Cohen et al. [59] found significantly negative pooled standardized mean difference (SMD) with substantial heterogeneity for total carotene (SMD −1.06, 95% CI −1.65–(−0.47), *p* < 0.01), β-carotene (SMD −0.40, 95% CI −0.72–(−0.08), *p* = 0.01) and lycopene (SMD −1.05, 95% CI −2.09–(−0.00), *p* = 0.05), whereas for α-carotene and lutein results were not significant. In conclusion, carotenoids role in the prevention of preeclampsia is inconclusive and there is the possibility of publications bias.

Oxidative stress is also noticed in GDM, which affects 1–14% of all pregnancies [60]. Plasma antioxidant capacity in women with GDM is lower as compared to health pregnant women, but no studies have been conducted to determine the existence of any differences in the plasma concentrations of carotenoids [61]. In an interventional study related to the role of carotenoids in protecting against oxidative stress it was found that taking supplements of carotenoids did not decrease the serum levels of hydroperoxides in pregnant women [62]. However, there was a significant difference in oxidative stress values between newborns born to mothers treated with lutein and newborns to mothers untreated at 2 h of life (*p* = 0.01). However, at 48 h, there was not a significant difference between the two groups. This study was conducted in a group of 24 women, 12 having GDM, who ingested supplements of 10 mg lutein and 2 mg zeaxanthin; however, there were no randomization or placebo controlling, and there were no demographic and anthropometric data of the participating mothers, hence the strength of evidence from these study is poor [62].

Preterm birth (<37 Hbd) has been shown to increase morbidity and mortality rate as well as perturbations in growth patterns. High level of oxidative stress has also been reported in premature birth even without complications or pathologies of pregnancy [63]. Approximately 30–40% preterm birth is caused by premature rupture of membranes (PROM). In addition, during the course of such pregnancy, high levels of biomarkers of oxidative stress in the amniotic fluid have been noticed [64]. Researchers from Canada noticed that women who give birth to preterm babies have lower serum concentrations of carotenoids and that the risk of premature birth decrease with the increased serum levels of α- and β-carotene, α- and β-cryptoxanthin, and lycopene [65]. The risk of premature birth may depend on the dietary habits of pregnant women. Investigators from USA have observed higher risk of preterm birth in women with lower intakes of β-carotene (delivery at <32 Hbd, OR 1.92, 95% CI 1.1–3.5) as well as α-carotene (delivery between 35 and 36 Hbd) [66]. In addition, a randomized controlled trial (RCT) during which subjects were given supplements of 2 mg lycopene daily from the first trimester of gestation revealed a weak but significant prolongation of gestational period in the subjects (37.72 ± 1.61 vs. 36.56 ± 2.2 Hbd, *p* < 0.05) [55]. Furthermore, researchers have noticed the importance of the consumption of sustainable diet (rich in fruits and vegetables, which are good sources of carotenoids) in the prevention of premature birth (hazard ratio (HR) 0.88, 95% CI 0.80–0.97) [67].

Newborns who are small for gestational age (SGA) are usually hypotrophic, and some of them are born from pregnancies with complications of IUGR. Both SGA and IUGR are characterized with high biomarkers of oxidative stress as well as low antioxidant capacity [68,69]. High maternal plasma concentrations of carotenoids (β-carotene, lutein and zeaxanthin, and α- and β-cyrptoxanthin) during pregnancy (24–26 Hbd) decrease the risk of giving birth to SGA babies (OR 0.64, 95% CI 0.54–0.78 for each increase in SD unit) [70]. Furthermore, mothers, who gave birth to newborns with IUGR have been shown to have lower levels of carotenoids in the serum and breast milk, but no differences were observed between the concentration of carotenoids in the serum and the skin of IUGR newborns and their healthy peers [71]. Considering this, the study by Sharma et al. [55] has shown that supplementation of lycopene at the dose of 2 mg daily during 16–20 Hbd can decrease the prevalence of the occurrence of IUGR (12% vs. 23.7% in the control group, *p* = 0.033). Researchers have also been interested in the impact of the intake of carotenoids and their status on birth parameters in healthy infants. Interestingly, studies on maternal carotenoid status as well as the content of these compounds in the cord blood did not show any of their impact on birth parameters of the newborn [69,72]. This was also revealed in an interventional study on the influence of β-carotene supplementation from the first trimester of gestation till the third month after birth (Randomized placebo-controlled trial, *n* = 13,709) [73]. However, this also study found unexplained small negative effect of β-carotene supplementation on birth weight (−18 g, *p* = 0.06), which need further examination in future studies [73]. As mentioned above, also Banerjee et al. [56] also found an adverse effect of carotenoids (lycopene) supplementation on the incidence of low birth weight, however the strength of this study is weak. Assessment of the influence of seasonal variations in diet during pregnancy showed a positive correlation between the intake of β-carotene in fourth month of pregnancy and head circumference at birth, but no relationship was found between seasonal variations in the intakes of nutrients and birth parameters [27].

## 5. Maternal-Fetal Transfer of Carotenoids

The absorption and transport of carotenoids is similar to that of fat. After absorption from the small intestine, carotenoids are initially transported via the lymphatic system, and later together with chylomicron remnants in the blood system, where they are transferred to the liver. In the liver, they undergo modifications as well as storage or are transported back to the circulatory system together with lipoproteins. Carotenes are transported with low-density lipoprotein (LDL), while xanthopylls, due to their higher polarity, are transported with high-density lipoprotein (HDL) and to a lesser extend with very low-density lipoprotein (VLDL). The affinity of carotenoids to different lipoprotein fractions and the number and proportions between their receptors in various issues determine the differences in the degree of saturation of different organs in carotenoids. For this reason, the highest amount of carotenes is found in the liver, testes and adrenal gland, where LDL receptor is primarily located, while xanthopylls are preferentially transported to the nervous tissue of the eye retina and the central nervous system due to the presence of HDL receptors [1,3,74]. Proteins, including albumin and lactoglobulin also participate in the transportation of carotenoids [75,76,77].

The exact mechanisms of the transfer, metabolism and utilization of carotenoids by the fetus remain to be elucidated. During pregnancy, the amount of maternal lipoproteins increases, which facilitates the uptake of carotenoids by the placenta. The dominating lipoprotein fraction in the cord blood is HDL, while LDL and VLDL are found in small amounts in the fetus blood. It has also been found that unlike in adults, HDL present in the cord blood participates to a greater extent in the transport of β-carotene than LDL (55% vs. 45%) [75,76,77,78]. In a mouse model study, it was noticed that administering β-carotene per os has the capacity to regulate the transcription and activity of microsomal triglyceride transfer protein (MTP) as well as placental apolipoprotein B (apoB), factors necessary for the biosynthesis of lipoproteins, which overall brings about an increase in the transfer of β-carotene to the placenta [79]. Deficiency of vitamin A has been shown to cause a decrease in the level of carotenoids in the placenta, which may be related to their poor transportation to this organ [80]. There is scarcity of data and research on the levels of carotenoids in various fetal tissues. Analysis of the concentration of carotenoids in the tissues of the vitreous body of aborted fetuses it was found that the maximum values of total albumin (1.42 mg) and carotenoids (276 ng) occurred approximately during 20–22 Hbd. Albumin and carotenoids concentrations peaked during weeks 17 and 16–17 (respectively) of prenatal development. Later, carotenoids concentrations gradually decreaserd, and by week 31 of gestation was below detection threshold [76].

## 6. Carotenoid Status in the Newborn

The most widely used biomarker of carotenoid status is maternal serum carotenoids as well as the level of these compounds in the cord blood. Most studies indicate that carotenoid status in the newborn directly depends on their level in the mother, which is usually several times lower. The concentration of carotenoids in the cord blood is at least several times lower than the level found in maternal blood [26,69,70,71,72,81,82,83,84]. Lower difference between the amount of carotenoids in the cord blood and maternal blood was observed for polar carotenoids (lutein, zeaxanthin, β-cryptoxanthin), which may be due the fact that during early life of the fetus till the first week of a child’s life, HDL is the dominating lipoprotein fraction [82]. Differences in the concentration of carotenoids, particularly β-carotene and other carotenoids that are precursors of vitamin A between the mother and the fetus may not be related to limitations in placental transfer of these compounds. This may be caused by intensive fetal metabolism, conversion of β-carotene to retinol, and later the storage of retinol esters in the liver, due to, among others, limited capacity to store β-carotene [85,86]. Henriksen et al. [71], who assessed macular pigment optical density (MPOD) and the concentration of carotenoids in the skin of 40 mother–child pairs (30 health and 10 with IUGR), obtained very interesting results. They found that both maternal and newborn plasma zeaxanthin correlated with MPOD in the infants (*r* = 0.59, *p* = 0.032 and *r* = 0.68, *p* = 0.007, respectively), but such a relation was not found for lutein, which is the main macula pigment in adults. This may be the result of the immature enzyme system, which is responsible for the conversion of lutein to meso-zeaxanthin found in the macula of the retina, which shows the importance of zeaxanthin in the development of macula pigment in infants.

Nutritional status of the newborn also depends on the week during which the newborn is delivered. This may be related to the dynamics of the fetus growth, which is highest during the third trimester of pregnancy. Analysis of the concentration of lutein and its metabolite 3’-oxolutein in the cord blood of a group of preterm and full-term newborns (*n* = 116, 33–42 Hbd) revealed that lutein concentration reaches its plateau at the beginning of the third trimester and starts to gradually decline from 37 Hbd, reaching its lowest value at 41–42 Hbd [87]. Other investigators found lower concentration of lutein in male newborns as well as newborns delivered from multiple pregnancy [34]. Smoking during pregnancy is another factor that decreases the concentration of carotenoids the cord blood, which was demonstrated in the case of β-carotene [26,33,88].

Furthermore, an increase in oxidative stress has been noticed during child delivery. Although the results of studies are equivocal, it has been observed that child birth via cesarean section, especially when planned, is related to higher oxidative stress [89,90]. Scaife et al. [26] did not find any differences in the concentration of β-carotene in relation to the type child birth, but Picone et al. [87] noticed a decrease in the level of lutein in the cord blood from newborns delivered via cesarean section.

## 7. Carotenoids in Breast Milk

The composition of breast milk depends on the lactation period, phase of single breastfeeding, time of day and frequency of breastfeeding during the day. Breast milk composition is also subjected to inter-individual variations as well as to the impact of other factors, including week of gestation, during which birth occurred as well as the frequency of breast emptying. The nutrient content of breast milk, including carotenoids, can be influenced by the dietary habits of the breastfeeding mother [91,92,93,94,95]. Fat in breast milk is highly subjected to changes and its content depends among others on lactation phase and phase of single breastfeeding. Colostrum contains relatively small amount of fat (about 2.6 g/100 mL), while mature milk has approximately 4.1 g/100 mL. In the beginning of the first breastfeeding, the amount of fat in the foremilk is negligible (about 1%), but the amount in hind-milk increases by even 9%. The amount fat in breast milk also changes depending on the time of the day; it is highest during the day and evening and lowest at night and in the morning [93,94,95,96].

Just like in the serum, the primary carotenoids in breast milk are β-carotene, lutein and zeaxanthin, lycopene, α-carotene and β-crptoxanthin [96,97,98,99,100,101,102]. Studies conducted in the US, Mexico and China showed that in all phases of lactation, the median content of carotenoids in breast milk was as follows: 114.4 nmol/L, 49.4 nmol/L, 33.8 nmol/L and 33.7 nmol/L for lutein, β-carotene, β-cryptoxanthin and lycopene, respectively [98]. In an earlier multinational investigation, Canfield et al. [97] found lower content of carotenoids with 62% of provitamin A carotenoids (α-carotene, β-carotene, and β-cryptoxanthin) accounting for all the carotenoids analyzed. The discrepancy in the results from various studies may be the result of different methods related to, among others, season for breast milk collection, methods of milk expression, methods used for fat extraction as well as dietary habits of population involved in the studies [98,102,103]. Similar to the fat content, hind-milk contains higher amount of carotenoids (by 25%) than the foremilk [102]. There is within day variability in breast milk composition, with higher fat content seen in the afternoon [100,102]. The concentration of carotenoids decreases with the duration of lactation (Table 2). Changes in the content of carotenoids between colostrum and mature milk can reach the values of 35.2–52.0% (zeaxanthin) and even 82.7–91.3% in the case lycopene [96,98,99]. The highest decline in the content of carotenoids occurs between the second and the fourth week of lactation, but stables between the fourth and the sixteenth week of lactation [98,104]. Less polar carotenes are subjected more to changes [96] as well as carotenoids, which are non-precursors of vitamin A (lycopene and lutein) [98].

Mother’s dietary habits, especially the consumption of fruits and vegetables, have a great impact on the content of breast milk carotenoids. The changes in the profile of the main carotenoids in breast milk observed in various studies could be due to the different dietary habits of the investigated populations [97,98]. Cena et al. [105] found high correlations between the intake of dietary lutein and its serum concentration (*r* = 0.94, *p* = 0.0001) as well as in breast milk (*r* = 0.86, *p* ≤ 0.0001). The content of milk carotenoids is easily altered their dietary intakes. It has been reported that a 3-day nutritional intervention with the use of carrot paste (15 mg β-carotene all-trans) or tomato paste (15 mg lycopene all-trans) by 26 women brought about an increase in the content of these carotenoids in milk even after the first day of intervention, but the highest concentration of lycopene (130% of the initial value) was found in subjects on the 4th day and a 200% increase for β-carotene after the 2nd day of intervention [106]. An increase in the breast milk content of lutein, zeaxanthin and β-carotene was also observed at the levels of 2.6×, *p* = 0.001; 2.7×, *p* = 0.001 and 1.7×, *p* = 0.049, respectively) after the use of chlorella supplements during 16–20 Hbd [107].

The concentration of carotenoids in breast milk is 10 and sometimes even 120 times lower than their plasma concentration and the strength of the relationship between them differs with relation to the investigated population or carotenoid. A study carried out in Italy showed that the strength of the relationship for lutein was *r* = 0.87, *p* ≤ 0.0001 [105] and *r* = 0.37, *p* ≤ 0.05 in a Brazilian study [108], while the correlations in a multinational study was found to be *r* = 0.45, *r* = 0.57; *p* ≤ 0.05 and *r* = 0.13, *p* > 0.05 for lutein, β-carotene and lycopene, respectively [98]. Differences have also been found in the ratio of milk carotenoids to their plasma content; higher ration was noted for more polar xanthopylls (0.12 ± 0.01 for lutein and zeaxanthin vs. 0.08 ± 0.001 for β-carotene [108]; 80% for lutein vs. 7–13% β-carotene and 4–5% for lycopene [98]. Randomized placebo-controlled trial revealed that a six-week supplementation with lutein (6 or 12 mg/day) caused and increase in its serum content by about 170% and 250% (*p* < 0.0001), respectively as well as in breast milk by 140% and 250% (*p* < 0.0001), respectively. The serum of lutein in infants exclusively breastfed was also found to be increased by 180% in the case of 6 mg/day and 330% after the ingestion of lutein in the dose of 12 mg/ day (*p* < 0.05) [109]. Lipkie et al. [98] showed the ratio of carotenoid in the serum of infants fed breast milk to their content in breast milk is variable and amounts to 133% for lutein, 560–600% for β-cryptoxanthin, and 270–300% for α-and β-carotene; the lower value for lutein can be attributed to its intensive turnover and uptake the newborn tissues.

Changes in breast milk content of carotenoids occur during the course of lactation, even in relation to milk volume of fat content. Changes in the profile of serum carotenoids as well as their content in plasma lipoprotein can also be observed. Differences in the ratio of serum carotenoids to their content in breast milk in relation to their polarity suggest that there is a different mechanism for the transfer of carotenoids to milk, which is independent of the transport of fat [96]. Breast milk constituents can be secreted in five different pathways, including four transcellular pathways (membrane pathway; transport via Golgi apparatus and secretion do milk through secretive cells via exocytosis; intercellular vesicle transport; and the transport of milk fat) as well as one paracellular pathway with help of tight-junction connections [98,115]. Milk fat is synthesized in, or on the surfaces of the rough endoplasmic reticulum membrane and accumulated in the form of droplets in the cytoplasm surrounded by milk fat globule membrane (MGFM). Droplets of variable size are transported to the apical pole of the cell through the cytoplasm and are secreted from the apical surface enveloped with cellular membrane. However, the transfer of carotenoids to breast milk can involve for example, preferential uptake by lipoproteins as well as intracellular transport. Breast tissue contains all types of lipoproteins, but mammary alveolar epithelium cells prefer the HDL fraction [98,99,116]. Furthermore, the transport of carotenoids into epithelial cells of the milk vesicles probably involves fatty acid transporting protein and cluster determinant 36 receptor (CD36), which is responsible among others, for the uptake of fatty acids as well as carotenoids, which precursors of vitamin A (β- and α-carotene as well as β-cryptoxanthin) in Caco-2 epithelial cells of the small intestine [98,99,115,117].

## 8. Infant Feeding Method and Infant Carotenoid Status

Due to humans inability to synthesize carotenoids de novo and fact that infant formulas often contain only trace amounts of those compounds as they are not routinely enriched in carotenoids, infant feeding method is an important determinant of infant’s carotenoid status, same as mother’s nutritional status [71,104,118]. Infants exclusively breastfed are characterized by better nutritional status as compared to infants mixed or artificially fed. Artificially fed infants after several months of receiving formulas have serum concentration of carotenoids several times lower as compared to values noticed after birth, and sometimes even lower than detection threshold [118,119,120,121]. This suggests the importance of enriching infant formulas with carotenoids, particularly with lutein, to improve or optimize the nutritional status of the newborns who are fed artificially. Studies on experimental formulas enriched with lutein demonstrated that they are well tolerated by newborns and improve their nutritional status [122]. A research carried out in infant rhesus macaques have also confirmed that feeding those animals with a mixture of feed enriched with carotenoids increases the saturation degree of their brain tissue in lutein but the increase in the amount of β-carotene, zeaxanthin and lycopene was negligible and below threshold of detection, what suggests the significance of lutein for the developing brain [123]. Due to lower bioaccessibility of lutein from infant formulas, it is necessary to consider a 4.5-fold increase in its concentration in relation to the levels found in breast milk [118,122,124].

## 9. Carotenoids and Infant Health and Development

There is an increasing number of studies on carotenoids role in infant health and development, due to widely documented health benefits and preferential uptake of carotenoids by fetus and breast milk [3,18]. The most important health benefits in infants may result from antioxidant properties of carotenoids and their role in visual and cognitive development [18,125].

### 9.1. Visual Development

Lutein, zeaxanthin and meso-zeaxanthin, isomer of zeaxanthin and lutein derivative, are uptaken by eye tissue, especially macula where they constitute the macular pigment [126]. The carotenoids concentrations in the eye tissue are not evenly distributed. The highest concentrations of carotenoids are observed in the central foveal region, and they decrease with the increasing distance from the fovea being 100-times lower in peripheral area. There are also differences in carotenoids proportions according to retinal region observed in the central foveal region dominate zeaxanthin (60% of total macular carotenoids; zeaxanthin:lutein ratio 2:1) and mezo-zeaxanthin and with the increasing distance from the central fovea the raise in the lutein concentration is observed [127,128,129]. Changes in macular pigment density and structure are also age-related. The peripheral retinal area is relatively mature after birth, but photoreceptors, fovea and MPOD intensively maturate in early childhood until at least 4–7 years of age [120,130,131,132]. Newborns have very low or undetectable mezo-zeaxanthin levels and opposite lutein:zeaxanthin proportion until two years of age, probably due to immaturity of enzymes involved in the conversion of lutein to mezo-zeaxanthin [71,127,128,129]. Moreover, premature infants have undetectable MPOD presumable for considerable immaturity of macula and carotenoid depletion due to shorter prenatal development [131]. It is also well known that infant MPOD correlates with maternal carotenoid status and after the birth infant feeding method is crucial for maintaining infant carotenoid status [71]. Breastfed infants have higher MPOD compared with those who are artificial fed, and recent study confirmed that early carotenoids exposition may be an important predictor of MPOD in adulthood [133,134].

Eye retina is very susceptible for oxidative damage due to extensive metabolic activity, high LC PUFA concentration and massive vascularity [130]. Newborns are even more vulnerable to such damages because of their immature autoregulation of blood flow within the choroid (what combined with an increased metabolic activity leads to hyperoxydation) as well as their more permeable lens that allow to pass higher amounts of energetic short-wave light [135,136]. Susceptibility of infant retina is reflected by rapid lipofucin accumulation during the first few years of life [137,138]. Prematures are even more vulnerable and extensive oxidative stress may lead to development of prematurity retinopathy [139].

Macular carotenoids protect retina by: (1) absorption of 40–90% of incident blue light, which protects the retina from photo-damages [140]; (2) antioxidant properties, i.e. a mixture of lutein, zeaxanthin and mezo-zeaxanthin can quench more singlet oxygen than individual carotenoid [141]; (3) anti-inflammatory and anti-apoptotic properties [142]; and (4) neuroprotective activity [143]. Lutein and zeaxanthin are also likely to support the transmission and processing of visual information by: (1) stabilization of microtubules in cytoskeleton [144]; (2) enhancing the gap junctional communication between glia and neuronal cells [145,146]; (3) improving visual parameters, including scoptopic noise and light scatter [147]; and (4) contribution in oxygen utilization from foveal [120,148]. There is also more scientific evidence supporting the crucial role of macular carotenoids in proper eye and visual development [130]. Animal model studies reveal that carotenoids are essential for proper retina development, including macula and retinal pigment epithelium density in fovea [149,150]. Additionally, proper visual development is crucial for optimal cognitive development of infant [18,151,152]. Besides, retinal lutein concentration is related to brain lutein concentration in primates [153], humans [154], as well as to cognitive performance in children and elderly [155,156].

### 9.2. Brain and Cognitive Development

Carotenoids are preferentially captured by the nervous tissue, and lutein constitutes even 59% of all carotenoids in infants, whereas only 31% in adults [123,155,157]. It has been shown that the most carotenoid is abundant in the brain area of the hippocampus, frontal cortex and occipital cortex, regions associated with cognitive process. In neurons, the highest lutein concentration is observed in cellular membranes and axon terminals, and the structure of neurons’ cell membranes amonal axonal projections depends on brain region in which they occur [144,158,159]. Lutein action in nervous tissue is related to: (1) improving intracellular communication [145,146,160]; (2) ability to modify the cellular membranes, including their fluidity, ion exchange, oxygen diffusion and stability [160]; (3) neuroprotective properties [161]; and (4) participation in metabolic pathways in brain [22]. The results of study conducted by Vishwanathan et al. [162] did not confirm the impact of infant feeding method on the brain content of carotenoids in full-term newborns. The brain of preterm newborns has lower concentrations of carotenoids as compared to full-term newborns [162,163].

Literature data have shown that carotenoids are important for cognitive performance, and its supplementation improves their cognitive performance in adults and elderly [18,155]. Recent study conducted among 55 exclusively breastfed infants from USA (82% of Caucasians) indicated that higher choline and lutein (whereas DHA and lutein had no effect) contents in breast milk at three (3.7 ± 0.63) months of age can improve the processes of cognitive functions in infants at six (6.1 ± 0.09) months of life, as assessed using an electrophysiology paradigm known as event-related potentials [20]. Observed association (study also revealed association for choline and DHA) may be caused by proposed mechanism of lutein and DHA transport to the brain via high-density lipoproteins, which are rich in phosphatidylcholine, as well as choline may have neuroprotective effect in itself [164,165].

### 9.3. Preterm Infants

Newborns are susceptible to oxidative stress, due to extensive metabolic activity and higher oxygen extrauterine environment [125]. Preterm infants are even more vulnerable to oxidative stress because of immature antioxidant defense system, associated disorders and invasive medical procedures [125]. Lutein may be protective against extender oxidative stress by improving biological antioxidant system and decreasing oxidation stress what has been shown in term infants who were supplemented with lutein at a dose of 0.28 mg at 6 and 36 h of life [166]. Lutein supplementation is well tolerated even by preterm infants [167] and may reduce the risk of developing diseases associated with premature birth such as retinopathy of prematurity (ROP), necrotizing enterocolitis (NEC) and bronchopulmonary dysplasia (BPA), however results were not statistically significant (Table 3).

Use of breast milk could influence on decrease of ROP risk (relative risk (RR) 0.39, 95% CI 0.17–0.92) as well as the risk of death before hospital discharge RR 0.27, 95% CI 0.08–0.96 [172], which may be related to high antioxidant activity of breast milk [173]. Recent study has revealed that donor milk had 18–53% decreased antioxidants status compared with maternal milk but in most cases still higher than infant formula [174].

### 9.4. Long-Term Studies in Infants and Children

The intake of carotenoids intake during pregnancy may also have long-term consequences for infants. Research conducted among mothers of children with sporadic retinoblastoma and health controls showed that inadequate intake of vegetables and fruits as well as lutein and zeaxanthin derived from such foods may increase the risk of sporadic retinoblastoma in children (OR 2.6, 95% CI 1.5–4.6) [19]. Carotenoids, especially provitamin A, may alter the various factors in the developing immune system, including T-cell proliferation or natural killer cell activity [175]. Study conducted by Litonjua et al. [17] revealed that maternal intake of lutein and zeaxanthin in the highest quartile compared with the lowest decreased risk of respiratory infections in two-year-old children (OR 0.56; 95% CI 0.37–0.85; *p* = 0.01) but there were no significant associations between wheezing or recurrent wheezing in the first two years of life. Results from Japanese study of 763 mother-infants dyads found that maternal β-carotene consumption during pregnancy in the highest quartile compared with the lowest decreased risk of infantile eczema, but not wheeze (OR 0.52, 95% CI 0.30–0.89) [21]. However, systematic review by Melo van Lent et al. [176] did not find any association between lutein intake or status and respiratory health in children. A cross-sectional study of healthy, well-nourished, children aged 5.75 years (*n* = 160) living in Vancouver, Canada investigating lutein intake and status did not confirm lutein role in cognition assessed by the Kaufman Assessment Battery (KABCC-II) and Peabody Picture Vocabulary Test (PPVT) [177]. Lack of lutein effect on cognitive performance may be caused by selection of well-nourished population, whereas largest functional effects of lutein may be the most significant for those with relative deficiency; selection poor biomarker (plasma concentration) for lutein in brain (MOPD would be better), as well probably not the most sensitive cognitive tests for measuring the effects of diet on brain development [178]. The Generation R Study (*n* = 2044 healthy Dutch children) did not support the hypothesis that lutein intake in early life (13 months of life) has beneficial role for later cardiometabolic health, anthropometrics and body measures at the age of six [179].

## 10. Safety of Carotenoids and the Intake Recommendations for Pregnant Women and Infants

It has been shown that carotenoids (lycopene, β-carotene, lutein) act as an antioxidant, but as oxygen pressure increases the effectiveness of carotenoids as an antioxidant decreases possible because of autooxidative processes [180,181]. This property may be responsible for results of few epidemiological studies which reported adverse health effect of high carotenoid intake with dietary supplements. ABTS clinical trial conducted in Finland among 29,133 50–69-year-old male smokers found that long-term (5–8 years) supplementation of 20 mg β-carotene per day caused 18% increase in incidence of lung cancers, and as a consequence 8% increased overall mortality [182]. In addition, later study has shown that this supplementation increased the post-trial risk of a first nonfatal myocardial infarction [183]. Other studies with higher dose of β-carotene (50 mg/day), but conducted among non-smoking participants did not shown that carotenoids supplementation lead to increase risk of cardiovascular morbidity or mortality [184,185]. The observed adverse health effect only in smoking participants may be associated with β-carotene oxidation by cigarette smoke, which lead to the formation of oxidation products of β-carotene [58]. Another, harmful, side effect of lutein supplementation is carotenodermia-a reversible condition characterized by yellowish discoloration of the skin [186]. To our knowledge, despite the one study in healthy low-risk pregnant women (2 mg lycopene/d since 15.7 ± 2.3 Hbd), there is no evidence of adverse health effects of high carotenoids supplementation or intake in pregnant women (even smoking) and newborns and infants, however none of studies conducted among these population were long term or using high dose of carotenoids.

There is no dietary intake recommendation for any of the carotenoids for any populations group, as they are not considered as essential nutrients. However, it has been argued that dietary intake recommendations for e.g., lutein and zeaxanthin should be established. In adults, there is strong evidence that intake of 6 mg lutein per day may be optimal for eye health, and there is no evidence of toxicity at intakes three times this dose in clinical trials [18,187,188]. There is no proposed intake recommendation of lutein for pregnant and breastfeeding women or infants, but, for infants, EFSA concludes that the concentration of 250 µg/L added lutein in infant formulae is safe [186]. During pregnancy, lactation and childhood diet rich in vegetables and fruits, as carotenoids source, should be recommended, due to the fact that intake of vegetables and fruits is associated with variety of health outcomes, as well there is no evidence that even high consumption of them is harmful [1]. For infants, exclusive breastfeeding up to six months and further continuation for up to two years and longer should be recommended, considering that breastfeeding leads to many health outcomes, and breast milk is a better source of carotenoids than formulae [189].

## 11. Conclusions

Carotenoids are nutrient with broadly documented antioxidant, anti-inflammatory and neuroprotective properties. Data from epidemiological, clinical and interventional studies have supported the associations between adequate intake of dietary carotenoids or its supplements and reduced risk of some chronic non-communicable diseases, as well as age-related decline in cognitive functions. However, among pregnant women, their role in the reduction of risk of pregnancy pathologies, as well pregnancy outcomes is inconclusive. Observational and few RCT studies contradict each other very often. In some cases, a beneficial effect of carotenoids on preeclampsia, preterm birth and infant birth parameters has been clearly observed; in others, little or no correlation between them has been found or, even, an inverse relationship has been reported. However, majority of conducted RCT studies were small and biased, so further research in these area are needed. Higher intake of carotenoids may be related to healthier diet and lifestyle, which may be beneficial in themselves. Lutein is one of the carotenoids which beneficial properties have been investigated more often. Lutein adequate intake during neonatal period may be particularly important as a result of its antioxidant activity and involvement in vision and nervous system development. In the early stages of life, visual stimuli are very important elements of stimulating the brain development, and in turn may be crucial for cognitive development of the child. Carotenoids status of newborns and infants, due to the lack of those components in most of infant formulas, depends on the nutritional status of the mother as well as the infant feeding method. It has been hypothesized that lutein and zeaxanthin during early childhood play a key role in the normal visual system development and also brain neurocognitive development. However, this has strongly been supported only in animal models. Due to the small number of available data or inconclusive results, it is necessary to continue research in this area. Further studies need to be performed to establish carotenoids role in early, as well subsequent, infant development and health are needed. RCT studies are highly recommended.

## Figures and Tables

**Table 1:** Mean intake of carotenoids intake during pregnancy.

Pregnancy Period	Study Group	Carotenoids		Source
		Type	Intake (µg/day)	
1 and 2 trimester	USA *n* = 1290 62.6% households with annual income of >$70,000 75.6% White, 10.4%, Black, 5.4% Hispanic, 4.9% Asian, 3.7% other ethnicity 9.5% smokers	dietary + supplements total β-carotene	4770.3 ± 2293.0	[17]
		dietary β-carotene	3863.5 ± 2036.5	
		dietary lycopene	7368.8 ± 3979.7	
		dietary lutein and zeaxanthin	2686.8 ± 1724.4	
		dietary α-carotene	878.0 ± 657.7	
		dietary β-cryptoxanthin	207.3 ± 130.9	
1–3 trimester	Poland *n* = 215 no information about socio-economic group white Caucasian	dietary β-carotene	4513.0 ± 3908.1	[29]
		dietary lycopene	4419.8 ± 3267.1	
		dietary lutein	2091.1 ± 1699.9	
9–20 Hbd ^1^	United Kingdom *n* = 774 26.6 ± 4.6 years 34% higher socio-economic group no information about ethnicity 40.4% smokers	dietary β-carotene	937 ± 789–1168 ± 890	[25]
		dietary total carotenoids	1323 ± 999–1843 ± 1125	
5–39 Hbd	Japan *n* = 763 30.0 ± 4.0 years 31.3% households with annual income ≥6,000,000 yen no information about ethnicity 90% European origin, 7% Maori, 3% other ethnicities	dietary β-carotene	2620.4 ± 1653.0	[21]
		dietary α-carotene	345.3 ± 277.0	
4 and 7 month of gestation	New Zealand *n* = 214 29.3 ± 4.4 years 72% higher socio-economic group	dietary β-carotene	1887–2510	[27]
34 Hbd	United Kingdom *n* = 1149 29.4 ± 5.5 years white Caucasian 45.5% smokers	dietary β-carotene	2302 ± 1861.6	[26]
		β-carotene supplements	90.88 ± 591.7	
		dietary + supplements total β-carotene	2394 ± 2001.2	

^1^ Hbd—week of gestation.

**Table 2:** The concentration of carotenoids in breast milk according to stage of lactation.

Lactation Stage	Study Group	Milk Collection Method	Carotenoid Concentration in Breast Milk (nmol/L)				Source
			β-Carotene	Lutein (L) and/or Zeaxanthin (Z)	Lycopene	β-Cryptoxanthin	
Colostrum	Germany; *n* = 21 30 ± 6 (20–39)	the total milk volume of one breast	423.4 ± 326.6	164.0 ± 84.9 L 33.2 ± 84.9 Z	508.9 ± 421.7	238.8 ± 156.1	[99]
	Cuba; *n* = 21 25 (19–30)	10–12 mL of primarily foremilk (in the morning)	125.7 ± 6.37 ^1^	67.9 ± 44.9 L ^2^ 9.7 ± 6.7 Z	137.3 ± 86.1 ^3^	61.1 ± 66.6 ^4^	[96]
	Italy; *n* = 21 33.9 ± 4.37 (24–42)	5–6 mL of milk	-	280 ± 220 L ^5^	-	-	[105]
Transitional milk	Cuba; *n* = 21 25 (19–30)	10–12 mL of primarily foremilk (in the morning)	44.2 ± 34.1 ^1^	44.5 ± 36.1 L ^2^ 8.6 ± 5.5 Z	44.2 ± 34.1 ^3^	24.8 ± 22.4 ^4^	[96]
Mature milk	Cuba; *n* = 21 25 (19–30)	10–12 mL of primarily foremilk (in the morning)	36.2 ± 17.2 ^1^	27.3 ± 16.4 L ^2^ 7.9 ± 7.7 Z	18.8 ± 2.7 ^3^	16.6 ± 12.7 ^4^	[96]
	Germany; *n* = 21 30 ± 6 (20–39)	the total milk volume of one breast	78.2 ± 46.2	88.1 ± 37.8 L ^2^ 19.5 ± 10.2 Z	59.8 ± 38.9 ^3^	60.6 ± 36.7 ^4^	[99]
	Brazil; *n* = 49 26.6 ± 6.3	the total milk volume of one breast	18.0 ± 2.0 ^5^	6.0 ± 1.0 L + Z ^5^	-	-	[108]
	Italy; *n* = 21 33.9 ± 4.37 (24–42)	5–6 mL of milk	-	110 ± 50 L ^5^	-	-	[105]

^1^, ^2^, ^3^, ^4^—results were converted from ng/mL units to nmol/L according to formula (ng/mL/molecular weight/1000); molecular weight: ^1^ β-carotene [110]; ^2^ lutein and zeaxanthin [111,112]; ^3^ lycopene [113]; ^4^ β-cryptoxanthin [114]; ^5^ results were converted from µmol/L units to nmol/L.

**Table 3:** The impact of carotenoids supplementation on oxidative stress and occurrence of prematurity disorders.

Study Group	Intervention	Assessed Outcomes	Results	Source
*n* = 77 GA ^1^ ≤ 34 (30.4 ± 2.3) 1415 ± 457 g Italy	RCT ^2^ L + Z ^3^ (0.5 + 0.02 mg/kg/day) vs. placebo	– total antioxidant status (TAS) plasma L and Z concentration	– no differences in TAS ↑ Z concentration at week 4 (*p* ≤ 0.05)	[168]
*n* = 203 GA ≤ 33 (29.6 ± 0.2) 1244 ± 32 g USA	RCT Formula with L + β-c + Ly (211 + 219 + 143 µg/L) vs. placebo control formula	– inflammatory status (C-reactive protein) electroretinography plasma carotenoids concentrations (compared also with breastfed infants)	– ↑ rod photoreceptor sensivity (6.1 vs. 4.1, *p* ≤ 0.05) ↓ inflammation status (*p* ≤ 0.001)	[169]
*n* = 114 GA ≤ 32 (28.8 ± 2.4) 1130 ± 330 g Italy	RCT L + Z (0.14 + 0.006 mg/kg/day) vs. placebo	– ROP ^4,5^ screening every 2 weeks	– ↓ ROP incidence in L/Z groups (19% vs. 27%, *p* > 0.05)	[23]
*n* = 63 GA ≤ 32 (29.9 ± 1.9) 1331 ± 415 g Italy	RCT L + Z (0.5 + 0.02 mg/kg/day) vs. placebo	– ROP screening plasma L and Z concentration every week	– no differences in ROP incidence ↑ L concentration at week 5 and Z at week 4 (*p* ≤ 0.05)	[167]
*n* = 229 GA ≤ 32 (30.1 ± 1.8) 1336 ± 417 Italy	RCT L + Z (0.14 + 0.006 mg/day)	– incidence of ROP, BPD ^6^, NEC^ 7^ till discharge or term corrected age	– ↓ ROP incidence in L/Z groups (6.2 % vs. 10.3%, *p* > 0.05) ↓ BPD incidence in L/Z groups (4.5% vs. 10.3%, *p* > 0.05) ↓ NEC incidence in L/Z groups (1.7% vs. 5.1%, *p* > 0.05)	[170]

^1^ GA—gestational age; ^2^ RCT—randomized controlled trial; ^3^ L, Z—lutein, zeaxanthin; ^4^ ROP—retinopathy of prematurity; ^5^ Criteria of International Committee for the Classification of Retinopathy of Prematurity [171]; ^6^ BDP—bronchopulmonary dysplasia; ^7^ NEC—necrotizing enterocolitis.

## Supplementary Materials

The following are available online at www.mdpi.com/2072-6643/9/8/838/s1, Table S1: Observational and Intervention Studies Examining Associations between Carotenoids during Pregnancy and Maternal or Infant Health Outcomes.
