# Supplementary material for: Health Effects of Carotenoids during Pregnancy and Lactation
Source: Nutrients. 2017 Aug 4;9(8):838. doi: 10.3390/nu9080838 (PMC5579631; doi:10.3390/nu9080838)
Supplement: Supplementary file 1 [file nutrients-09-00838-s001.pdf]

Table S1. Observational and intervention studies examining associations between carotenoids during pregnancy and maternal or infant health outcomes

Study type	Study group	Observation or intervention	Assessed outcome	Results	Source
**Observational studies**					
Prospective cohort study	n=67 Norway, Australia, USA<br>nondiabetic (n=20) and 1 type diabetic (n=47)<br>31.7 ± 4.6y, 28.5 ± 5.6<br>non-Hispanic	maternal serum carotenoids (α-C, β-C, Ly, L[1]) in 1, 2, 3 trimesters of pregnancy	subsequent PE+[2]	-↓ 45% α –C and 53% β-C plasma concentrations t 3 trimester in PE+ diabetic compared with PE-[3] diabetic ($p<0.05$)	[49]
Case-control study	n=304 Peru<br>PE+ n=125, PE- n=179<br>26.6 ± 0.6y,-26.3 ± 0.5y<br>n=271 Inca Indian ancestery<br>n=48 <12 y of education	maternal serum carotenoids (α-C, β-C, Ly, L·, Z, β-Cr[4]) in 36.0 – 37.3 Hbd[5]	differences in carotenoids level between PE+ and PE-	-no differences	[50]
Case-control study	n=359 Zimbabwe<br>PE+ n=173, PE- n=186<br>25.6 ± 6.4y, 26.6 ± 5.8y	maternal serum carotenoids (α-C, β-C, Ly, L·, Z, β-Cr) at 12 – 72h postpartum	differences in carotenoids level between PE+ and PE-	↓ 45%PE+ risk for women in the highest quartiles for α-C, β-C, L, Z, β-Cr:<br>- β-C OR[6] 0.50 (95%CI[7] 0.25-1.00)<br>- α -C OR 0.53 (95%CI 0.26-1.05)<br>- β-Cr OR 0.47 (95%CI 0.19-1.11)<br>- L OR 0.46 (95%CI 0.19-1.11)<br>- Z OR 0.48 (95%CI 0.20-1.16)	[41]
Case-control study	n=5337 Canada<br>PE+ n=111, PE- n=441<br>29.3 ± 5.7/5.4y<br>18 and 15% high school or less, 26 and 39% university graduate or more<br>75 and 82% North America/Europe/Australia	maternal serum carotenoids (α-C, β-C, Ly, L·, β-Cr, α-Cr[8]) in 24 – 26 Hbd	subsequent PE+	↓ PE+ risk:<br>- L OR 0.60 (95%CI 0.46-0.77)<br>- Ly OR 0.93 (95%CI 0.75-1.16)<br>- carotenoids OR 0.82 (95%CI 0.62-1.07)<br>- L OR 0.53 (95%CI 0.35-0.80) – early onset PE<br>- L OR 0.62 (95%CI 0.47-0.82) – late onset PE	[48]

	5 and 11% family income<15000$/y				
Cohort study	n=23 423 Norway<br>5.4% PE+<br><20y 3.8, 20-29y 58.1%, 30-39y 37.2%, ≥40y 0.9%<br>≤10y of education 4%, ≥16y 21%<br>5.4% daily smokers	dietary pattern at 17-22 Hbd (FFQ[9])	subsequent PE+	↓ risk for dietary pattern rich in vegetables and fruits:<br>- OR 0.72, 95%CI 0.62-0.85)	[51]
Case-control study	n=650<br>n=207 preterm birth, n=443 term<br>20-34y 78 and 83%<br>high school or less education 14 and 18%<br>family income per year <15000 12 and 14%	maternal serum carotenoids (α-C, β-C, Ly, L, β-Cr, α-Cr) in 24 - 26 Hbd	subsequent spontaneous preterm birth	↓ risk for the highest quartile compared with the lowest:<br>- β-C OR 0.4 (95%CI 0.2-0.7)<br>- α -C OR 0.5 (95%CI 0.3-0.9)<br>- β-Cr OR 0.6 (95%CI 0.3-1.1)<br>- L OR 0.6 (95%CI 0.4-1.1)<br>- Ly OR 0.6 (95%CI 0.4-1.1)	[65]
Case-control study	n=5738 USA<br>n=471 preterm birth, n=5267 term<br>26.21 ± 6.61, 27.55 ± 6.07<br>46.6 – 59.6% Non-Hispanic white<br>16-19% education < high school<br>15.5-25.5% smoking<br>4-6.9% gestational diabetes	maternal dietary intake year before pregnancy	subsequent preterm birth	↑ risk for the lowest quartile compared with the highest:<br>- β-C OR 1.9 (95%CI 1.1-1.7) - 32-34 Hbd<br>- α -C OR 1.4 (95%CI 1.1-1.9) – 35-36 Hbd	[66]
Prospective cohort study	n=66000 Norway<br>5.3% preterm birth	dietary pattern during pregnancy (FFQ)	subsequent preterm birth	↓ risk for the prudent dietary pattern (rich in vegetables and fruits):<br>- HR[10] 0.88 (95%CI 0.80-0.97)	[67]

	52.5%, ≥40y 2.0% <20y 1.0, 20-29y 44.5%, 30-39y<br><13y of education 31%, ≥17y 25%<br>5.4% daily smokers<br>singleton pregnancies				
Case-control study	n=996 Canada<br>n=324 SGA[11], n=672 controls<br>29.01 ± 5.74 – 29.07 ± 5.16y<br>19.8 and 15.5% high school or less, 35.5 and 38.1% university graduate or more<br>76.9 and 79.9% North America/Europe/Australia<br>13.6 and 10.9% family income<15000$/y<br>15.3 and 26.9% smokers	maternal serum carotenoids (β-C, Ly, L, β-Cr, α-Cr) in 24 - 26 Hbd	risk of SGA	- Ly OR 0.89 (95%CI 0.77-1.03)<br>- carotenoids OR 0.64 (95%CI 0.54-0.78)	[70]
Cross-sectional study	n=200 Germany<br>32.3 ± 5.0 y<br><br>no data about maternal characteristic<br>Weeks of labour 39.1 ± 1.5	maternal serum carotenoids (α-C, β-C, Ly, L, Z, β-Cr) at delivery	birth parameters	- no associations	[69]
Prospective cohort study	N=251 Poland<br>28.26 ± 3.72 y<br>64% education beyond high school<br>21.5% environmental cigarette smoke exposure<br>Weeks of labour 39.61 ± 1.2	maternal serum carotenoids (α-C, β-C, Ly, L, Z, β-Cr) at delivery	birth parameters	- no associations	[72]

Retrospective observational study	Italy n=24<br>gestational diabetes mellitus, insulin therapy<br><br>no data about maternal characteristic	10mg L +2mg Z/d (n=12) since 28th Hbd for delivery<br>vs.<br>no supplementation (n=12)	oxidative stress in mother and newborn (TH[12] value)	- no difference in maternal oxidative stress<br>- ↓ oxidative stress at 2 h of life in infants (26.5 ± 5.74 vs. 60.4 ± 21.6 TH value, p=0.01)<br>- no difference in oxidative stress at 48-60 h of life (71.0 ± 27.2 vs. 74.1 ±24.8 TH value, p=0.84)	[62]
**Intervention studies**					
RCT	India n=251<br>low-risk, healthy<br>21.8 - 22.6 y<br>8.6 – 9.3 y of education<br>singleton pregnancy	2mg Ly/d (n=116)<br>since 16-20 Hbd<br>for delivery<br>vs.<br>placebo (n=135)	subsequent PE+, fetal outcomes	- ↓ PE+ (8.6 vs 17.7% cases, p=0,043)<br>- ↓ incidence of IUGR[13] (12% vs. 23.7%, p=0.033)<br>- ↑ birth weight (2751.2 ± 315.8 vs. 2657.3 ± 444.3, p=0.049)<br>- ↑ gestation weeks (37.7 ± 1.6 vs. 36.6 ± 2.2, p<0.05)	[55]
RCT	India n=159<br>low-risk, healthy<br>24.2 – 24.6 y<br>singleton pregnancy	2mg Ly/d (n=77) vs. placebo (n=82)<br><br>since 12-20 Hbd for delivery	subsequent PE+, fetal and maternal outcomes	- no differences in PE+<br>- gestational weeks<br>- birth weight<br>- ↑ incidence of preterm labour (10.4% vs. 1.22, p=0.02)<br>- ↑ incidence of low birth weight (22.1% vs. 9,8%, p=0.05)	[56]
RCT	n=44 India<br>high-risk of PE+,<br>18-23y n=26, 24-28y n=10, >28y n=8,<br>singleton and multiple (n=3) pregnancies	2mg Ly/d (n=20) vs. no supplementation (n=24)	subsequent PE+ development, fetal outcomes	- no differences in PE+<br>- ↓ incidence of IUGR (5% vs. 33.3%, p=0.027)	[57]

		since 14-28 Hbd for delivery			
RCT	N=13709 Bangladesh Rural area	vitamin A 7000 μg Res[14]/d vs. β-C 42mg/d (7000 μg REs/d) vs. placebo since 1 trimester for 3 mo postpartum	pregnancy duration and birth parameters	- no significant differences	[73]

[1]- α-C - α-carotene, β-C - β-carotene, Ly - lycopene, L – lutein; [2]PE+ – with preeclampsia; [3]PE- – without preeclampsia; [4]Z – zeaxanthin,, β-Cr - β-cryptoxanthin; [5]Hbd – week of gestation; [6]OR – odds ratio; [7]CI - confidence interval; [8]α-Cr - α- cryptoxanthin; [9]FFQ – Food Frequency Questionnaire; [10]HR – hazard ratio; [11]SGA – small for gestational age; [12]TH – total hydroperoxides; [13]IUGR – Intrauterine Growth Restriction; [14]REs – retinol equivalents.
